# Share and share alike: the role of Tra1 from the SAGA and NuA4 coactivator complexes

**DOI:** 10.1080/21541264.2018.1530936

**Published:** 2018-10-30

**Authors:** Alan C. M. Cheung, Luis Miguel Díaz-Santín

**Affiliations:** a Department of Structural and Molecular Biology, University College London, Institute of Structural and Molecular Biology, London, UK; b Institute of Structural and Molecular Biology, Biological Sciences, Birkbeck College, London, UK

**Keywords:** Tra1, SAGA, NuA4, histone acetylation, coactivator, activator

## Abstract

SAGA and NuA4 are coactivator complexes required for transcription on chromatin. Although they contain different enzymatic and biochemical activities, both contain the large Tra1 subunit. Recent electron microscopy studies have resolved the complete structure of Tra1 and its integration in SAGA/NuA4, providing important insight into Tra1 function.

## Introduction

Complex programs of transcription are established by DNA-specific transcription factors (activators) which enable recruitment of coactivators to target genes. Coactivators then stimulate transcription by assembling the RNA Polymerase II (Pol II) Pre-Initiation Complex (PIC) and/or catalyzing chromatin modifications. As coactivators are far less numerous than activators, they act as integrative hubs within the activation process. The mega-dalton SAGA and NuA4 coactivator complexes are required for transcription on chromatin; SAGA is a 19-subunit complex that catalyzes histone H3 acetylation, H2B deubiquitination and interacts with the PIC via TBP, whereas NuA4 is a 13-subunit complex that catalyzes histone H2A, H2A.Z and H4 acetylation and also interacts with the Pol II CTD during transcription elongation [1]. They also have diverse and distinct non-transcriptional activities; NuA4 has an important role in DNA repair, whereas SAGA participates in telomere maintenance and mRNA export. Despite these functional and mechanistic differences, both complexes have integrated the very large Tra1 (yeast) or TRRAP (human) subunit [2,3], a 433 KDa protein that accounts for ~ 30% of their mass and is a direct target of multiple activators, interacting with VP16, c-Myc, E2F and E1a in human cells, and with metabolic activators Gcn4, Hap4 and Gal4 in yeast [4–7]. Hence Tra1 enables the targeting of SAGA/NuA4 activities to the genome during transcription activation. In addition to activator-mediated recruitment, SAGA/NuA4 harbors a wide variety of domains that interact with modified histone tails e.g. Tudor-, bromo- and chromo-domains in Sgf29 (SAGA), Gcn5 (SAGA), and Eaf3 (NuA4) respectively, providing additional means of SAGA/NuA4 recruitment to specific loci.

**Figure 1.**
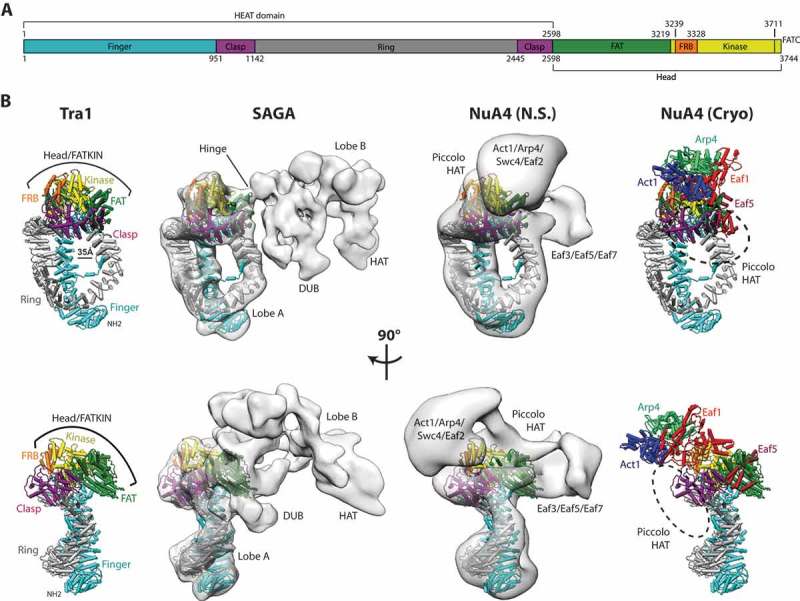
(a) The domain organization of Tra1. The HEAT domain is split into three subdomains named Finger, Ring and Clasp. The C-terminal FAT, FRB, Kinase and FATC domains are collectively referred to as the Head. Residue numbering is shown for S. cerevisiae Tra1. (b) The structure of Tra1 and its location within the SAGA and NuA4 complexes. Tra1 is shown in cylinders representation and color matched to (a). The 35Å aperture between finger and ring subdomains is highlighted by a double headed arrow. The upper and lower row of panels are related by a 90 degree rotation of each structure about the vertical axis, maintaining the same orientation and coloring of Tra1 within each row. Running left to right, the panels show the structure of isolated Tra1 [11], its position within SAGA [12], and within two different NuA4 reconstructions (negative stain (N.S.) [20] and cryo-EM [21]). The locations of the catalytic SAGA histone acetyltransferase (HAT) and histone deubiquitinase (DUB) modules are indicated. The location of individual NuA4 subunits and its catalytic module (Piccolo HAT) are also indicated.

Interestingly, Tra1 also belongs to the Phosphatidylinositol-3 kinase-related kinase (PIKK) family, a group of highly conserved protein kinases that includes TOR, ATM, ATR, DNA-PKcs and SMG-1. PIKKs are large proteins with domains named HEAT, FAT, FRB, Kinase and FATC arranged sequentially from N- to C- terminus (Figure 1(a)). The HEAT and FAT domains are composed of HEAT and TPR helical repeats respectively, whereas the FRB and FATC domains are small helical domains that are closely associated with the kinase domain. PIKKs are central regulators of cellular growth, proliferation and stress responses, phosphorylating multiple targets in DNA repair, metabolic control and mRNA surveillance. Intriguingly, Tra1 is the only pseudokinase member of the PIKK family due to loss of key catalytic residues in the active site, but the kinase domain is nevertheless important given its sensitivity to mutations which often renders cells inviable [8,9]. Similarly, Tra1 is an essential protein as its deletion causes lethality in budding yeast and knockdown of mammalian Tra1 is embryonic lethal. One important exception is in fission yeast which contains two paralogs named Tra1 and Tra2 that specifically associate with SAGA and NuA4 respectively; loss of SAGA-associated Tra1 is viable, whereas NuA4-associated Tra2 is essential [10], indicating that its role in NuA4 is critical for cell function but is dispensable in SAGA.

## The structure of Tra1

Despite its conservation and importance, elucidation of Tra1 function has been hampered by its large size and previous studies have generally relied on genetic experiments. However, recent advances have been made in electron microscopy (EM) studies of Tra1 in isolation and as part of SAGA/NuA4, shedding new light onto its functions and mechanisms in multiple complexes. We determined a 3.7 Å structure of the isolated Tra1 protein from *Saccharomyces cerevisiae* [11], concurrently with the Schultz group (IGBMC, Strasbourg) who solved a 5.9 Å structure of *Pichia pastoris* Tra1 as part of a larger reconstruction of the SAGA complex [12]. The structure is dominated by alpha solenoid folds whose organization resembles a “diamond ring”, where the HEAT domain forms the band, the FAT domain forms the setting and the remaining domains represent the center stone. This analogy was used to define regions of *S. cerevisiae* Tra1 termed Finger, Ring, Clasp, FAT, FRB, Kinase and FATC where the Finger, Ring and Clasp are subdivisions of the HEAT domain (Figure 1(a,b)). The N-terminal Finger forms a solenoid that is packed against a larger, circular solenoid comprized of the Ring and Clasp regions which form a closed loop, and this juxtaposition of Ring and Finger creates an unusual 35 Å wide aperture between them (Figure 1(a)). The remaining C-terminal FAT, FRB, Kinase and FATC domains collectively form a globular region named “Head” (often referred to as FATKIN (FAT plus KINase) in other PIKKs) that sits on an interface formed by the HEAT domain, and adopts a conformation that is highly consistent between all PIKKs: the TPR repeats of the FAT domain form a tightly curved arch within which the FRB, Kinase and FATC domains are nestled. Although the HEAT domain conformation varies dramatically between different most PIKKs, the Tra1 HEAT domain bears a striking resemblance to the DNA-repair factor DNA-PKcs which also forms a similar “diamond-ring” conformation [13], and deviates only within the Finger which is more curved in DNA-PKcs. Given the low sequence similarity, this was unexpected and may point toward a role for Tra1 in DNA repair which has been inferred from previous studies [14,15].

Alpha-solenoids typically form highly flexible protein folds and it was expected that Tra1 would have similar properties given its abundance of helical repeats. However, little structural heterogeneity was observed during Tra1 image processing, and the final reconstruction contained a majority (44%) of the collected particles, clearly indicating that Tra1 forms a very rigid structure. The topology of Tra1 explains this rigidity, as the organization into a diamond ring conformation effectively braces or “knots” the solenoids together, reducing their flexibility. However, local resolution analysis indicates that the N-terminal region of the Finger is more mobile, in concordance with a less restrained position.

## SAGA/NuA4 integration compared

Recent electron microscopy studies have visualized the SAGA and NuA4 complexes at a resolution sufficient to place the Tra1 subunit, and allows the integration of Tra1 to be compared and contrasted. At the resolutions attained, the structure of isolated Tra1 appears identical to that within SAGA and NuA4, adopting the same overall conformation regardless of complex integration. Cryo and negative stain EM reconstructions of complete SAGA [11,12,16] from both P. pastoris and S. cerevisiae reveal a bi-lobed structure in which Tra1 occupies Lobe A, with the remaining SAGA subunits located in the opposite Lobe B (Figure 1(b)). Interaction between these lobes are limited to a small contact point called the “Hinge” [12] which maps to the FAT domain on the Tra1 side. Crosslinking-coupled to mass spectrometry (CXMS) studies identified ~ 16 crosslinks between Tra1 and SAGA subunits Ada1, Spt20, Spt3, Spt7, Sgf73, Taf6 and Taf12, making them potential candidates that contact Tra1 [17,18] on the opposite side of the hinge. Collectively, these studies show that Tra1 is peripherally attached to SAGA and not a scaffold around which the complex assembles, and suggested by earlier work which showed that deletion mutants of Ada1 and/or Spt20 can dissociate Tra1 [19], and that SAGA can be purified intact without Tra1 after its deletion in S. pombe [10]. Conversely, recent CXMS data [20], negative stain [20] and Cryo-EM [21] reconstructions of NuA4 reveal a more extensive interface to Tra1 that may contribute to NuA4 structural integrity. A 27Å-resolution negative stain reconstruction of glutaraldehyde crosslinked NuA4 revealed a T-shaped architecture (Figure 1(b), lower panel) in which Tra1, Eaf1 and the catalytic subcomplex Piccolo (Esa1/Epl1/Yng2/Eaf6) [22] occupies the central stem, one arm contains subunits Swc4/Act1/Arp4/Yaf9 that are shared with the SWR1 complex [23] and the opposing arm contains the TINTIN [24] (Eaf3/Eaf5/Eaf7) subcomplex. This architecture is consistent with CXMS analysis which identified 40 intermolecular crosslinks between the C-terminal Head region of Tra1 and NuA4 subunits Eaf1, Eaf5, Esa1, Arp4, Epl1, Yaf9 and Swc4 [20]. A subsequent cryo-EM study reconstructed two subcomplexes of NuA4 without crosslinking at 4.7 and 7.6 Å-resolution, termed TEEAA (for Tra1/Eaf1/Eaf5/Actin/Arp4) and TEEAA-Piccolo respectively, where a partial atomic model was built for the former (Figure 1(b)). Unlike the negative stain study, the Cryo-EM reconstructions were L-shaped rather than T-shaped, with the long stem containing Tra1, Eaf5 and Piccolo, and the short arm containing the remaining subunits. This difference is presumably due to loss of TINTIN subunits Eaf3/Eaf7, and SWR1 shared subunits Swc4/Yaf9 that were not resolved in the cryo-EM reconstruction, but despite their difference in composition, are broadly in agreement in their positioning of their common NuA4 subunits which are clustered around the Head of Tra1 in two lobes (Figure 1(b)). One exception is the position of Piccolo; although both studies associate Piccolo with Tra1, its precise location differs as it is located at the Head in negative stain vs. the Ring in Cryo-EM (Figure 1(b)), and may represent inherent flexibility in its attachment to NuA4. Nonetheless, the central position of Tra1 within NuA4 suggests that Tra1 may form a scaffold around which the NuA4 complex is assembled, a function that is also ascribed to Eaf1 [25]. A scaffolding function for NuA4 may partly explain why the NuA4-associated Tra2 paralog in fission yeast is essential, as its loss may destabilize NuA4-complex integrity, unlike the SAGA-specific Tra1, which can be deleted from fission yeast without affecting SAGA integrity or cell viability. However, NuA4 destabilization per se may not be the complete cause of ΔTra2 lethality, as deletion of scaffolding subunit Eaf1 in budding yeast also destabilizes the NuA4 complex but produces viable cells, albeit with compromized growth [25].

Superposition of SAGA and NuA4 reconstructions via their Tra1 subunits shows severe clashes between the SAGA Lobe B and the NuA4 TINTIN subcomplex, preventing their simultaneous association to the same molecule of Tra1. Thus contacts by SAGA and NuA4 are made to the same surfaces of Tra1, largely within C-terminal Head which is consistent with the vast majority of intermolecular crosslinks identified by XLMS. Although both SAGA and NuA4 make contacts to the FAT domain, NuA4 makes additional contacts to the Kinase and FATC domains, enlarging its contact area when compared with SAGA, and reflected in the ~ 2–3-fold greater number of XLMS crosslinks obtained. But both interfaces are small when compared to the total surface area of Tra1, and leave almost the entire HEAT domain accessible. As biochemical and genetic studies implicate the HEAT domain as important for activator interactions, its equivalent presentation in both coactivators suggest that activators cannot discriminate between SAGA and NuA4 when targeting the Tra1 HEAT domain.

## Activator targeting

The most frequently described molecular function of Tra1 is as an activator target, enabling the activities of SAGA and NuA4 to be directed to specific loci. This property is conserved across all eukaryotes, with numerous genetic and in-vitro experiments describing interactions of Tra1 or TRRAP with a wide range of transactivation domains (TADs) contained within yeast and human activators [4,9,26–30]. Many of these studies also identify specific regions or residues of Tra1 that affect activator binding and transcription that may represent direct interaction sites. When mapped to the structure, these residues are distributed across Tra1 but predominantly localize to the HEAT domain, particularly at the intersection between the N-terminal half of the Finger and the Ring subdomains [11] (Figure 1(a)). Activators that target this region include yeast Gal4 [28], Gcn4 and Rap1 [9], and human p53 [26] and c-Myc [30], suggesting that this region of Tra1 is a key interaction platform for activators, although an important exception is VP16 which interacts with the C-terminal half of Tra1 [4]. Importantly, many of these Tra1 mutations affecting activator-stimulated transcription are selective for specific activators; mutations that affect activation by Gal4 [28] are distinct from those affecting Rap1 and Gcn4 [9] and are also located on different regions of the HEAT domain, indicating the presence of multiple and distinct activator binding sites. Given that activators often act combinatorially [31], multiple TAD binding sites could provide a simple mechanism for Tra1 to integrate signaling from multiple activators e.g. by different activators binding Tra1 simultaneously and/or competitively. Alternatively, the presence of multiple TAD binding sites could be used to bind multiple TADs within a single activator to increase avidity, as described between activator Gcn4 and mediator subunit Med15 [32]. In addition to Tra1, SAGA subunits Ada1, Gcn5, Taf6 and Taf12 also make direct interactions with activators VP16, Myc, Gcn4 and Gal4 [7,33–35] which may provide redundancy in recruitment, explaining why the loss of Tra1 functionality from SAGA is viable [10]. To our knowledge, no direct activator targets other than Tra1 have been described for NuA4, hence Tra1 might be the only means by which NuA4 is recruited to promoters by activators. Together with its scaffolding position within the complex, this may explain why loss of the NuA4-specific paralog Tra2 is lethal in S. pombe [10]. Nevertheless, for most other eukaryotes, the integration of the same protein into both SAGA and NuA4 may allow a single activator to recruit a wide variety of chromatin modifying activities to the genome.

## Are there additional functions for Tra1?

The recent EM studies of Tra1, SAGA and NuA4 have provided key mechanistic insights into Tra1 function but many questions still remain unanswered, particularly the reason for its common integration into both complexes. Given its role in binding TADs, the simplest explanation is that Tra1 allows a single activator to recruit both SAGA and NuA4, and functions as a passive bridge between activators and coactivators. However, given Tra1’s size, rigidity and conservation, its relationship to the PIKK family and its sensitivity to mutagenesis, we speculate that Tra1 may have additional functions beyond activator targeting. An indication of more complex behavior within SAGA and NuA4 comes from mutants of Tra1 that reduce histone acetylation activity without affecting recruitment or complex assembly [8,9], and is suggestive of communication between Tra1 and the catalytic HAT subunits. Given the differing architecture and enzymatic activities of SAGA/NuA4, and the limited contacts between SAGA and Tra1, an allosteric mechanism mediated by Tra1 that directly modulates catalysis in both complexes is hard to envisage. Hence Tra1 effects on HAT activity could represent a novel role in recruiting or orienting nucleosome substrates to the catalytic subunits of SAGA/NuA4. Tra1 may also have potential functions in DNA repair [15], given its structural similarity to DNA repair factor DNA-PKcs, its interaction with the MRN repair complex [14] and its requirement for cell cycle progression through the mitotic checkpoint [36]. With the advent of integrative structural biology and improvements in recombinant expression technologies, we are now able to study Tra1/SAGA/NuA4 in vitro, and will explore these aspects in the near future.
